# Optimizing patient and public involvement (PPI): Identifying its “essential” and “desirable” principles using a systematic review and modified Delphi methodology

**DOI:** 10.1111/hex.12618

**Published:** 2017-09-19

**Authors:** Rebecca L. Baines, Sam Regan de Bere

**Affiliations:** ^1^ Collaboration for the Advancement of Medical Education Research & Assessment University of Plymouth Plymouth UK

**Keywords:** delphi, health care, patient and public involvement, qualitative

## Abstract

**Background:**

There is international interest in the active involvement of patients and the public. However, consensus on how best to optimize its application is currently unavailable.

**Objective:**

To identify and assess the underlying principles of patient and public involvement (PPI) in health and social care services, research, education and regulation across medicine, dentistry and nursing.

**Design:**

A four‐phase methodology: (i) an extensive systematic review of published and grey literature; (ii) inductive thematic analysis of review findings; (iii) development of best practice principles; and (iv) consensus testing of identified principles using a modified Delphi methodology.

**Setting and participants:**

Twelve systematic reviews and 88 grey literature publications were reviewed leading to the unique identification of 13 principles later assessed by 18 PPI experts.

**Results:**

Essential consensus (>75% agreement) was obtained for nine principles reviewed. Working in equal partnership and sharing information achieved the highest consensus rates: 16/17 essential 94.1%; 1/17 desirable 5.8%. The four remaining principles that failed to reach essential consensus were categorized as desirable by expert respondents. No principles were considered irrelevant. No alternatives were suggested.

**Discussion:**

Expert respondents suggest essential principles must be achieved to optimize PPI best practice. To advance PPI practice, desirable principles should also be aspired to wherever possible.

**Conclusions:**

This study's innovative approach advances existing knowledge by providing previously unavailable consensus about PPI best practice. Research findings hold important theoretical and practical implications for educators, regulators, researchers and practitioners looking to effectively work together.

## 1 | INTRODUCTION

The practice of patient and public involvement (PPI) is multidisciplinary in nature and international in scope.[1-3] Although other definitions are acknowledged, for the purposes of this study, PPI is defined as the "active involvement of patients, service users, carers or family members in activities done *with* or *by*, rather than *to*" them.[4]

Often influenced by mandatory policies or requirements,[5,6] PPI has been applied to a variety of settings including health-care services,[7] regulation,[8] education[3] and research.[1] However, in spite of its widespread application and well-documented benefits including enhanced safety and quality of care,[9-11] consensus on how best to optimize its application is currently unavailable.

One explanation for this discrepancy is a lack of a common language to share PPI practice.[3] For example, in both policy and practice, the term PPI is not universal in its application or definition.[12,13] Such issues have arguably led to an overwhelming number of circular debates held primarily amongst academics with limited patient involvement,[13] causing the fundamental principles required for individuals to effectively work together (the central tenet of all PPI initiatives regardless of its "label," context or topic of interest) to be largely overlooked.[13]

Whilst discussions about the semantic intricacies of PPI are important in certain contexts, as argued by Brett et al[11] it is equally important to identify principles that can best enhance its potential impact.[8,11,14,15] Given that PPI now forms a central tenet of most policy requirements in health and social care services, it is imperative that consensus is achieved on the principles underpinning it. In spite of international interest, there is as yet limited research that explores PPI best practice, and less still that has been rigorously assessed or evaluated from a patient perspective.[16]

This research, therefore, aims to inform PPI best practice across historically fragmented disciplines through the innovative application of a systematic review and modified Delphi methodology. The research is unique not only in its scope and methodology, but also in its inclusion of both an evidence-informed and patient/public expert perspective. It contributes to, and advances existing knowledge by exploring areas of consensus and conflict, the central premise of Delphi methodologies, across the inter-related but until now disparate settings of: health and social care services, education, research and regulation across medicine, dentistry and nursing.

## 2 | METHODS

This research draws on a four-phase methodology: (i) an extensive evidence review of published and grey literature exploring PPI across the settings of health and social care services, research, education and regulation in medicine, nursing and dentistry; (ii) inductive thematic analysis of review findings; (iii) development of PPI principles based on thematic analysis findings; and (iv) consensus testing of identified principles using a modified Delphi technique comprised of three rounds. Similar to the original, the modified Delphi technique begins with a pre-defined set of carefully selected items.[17] For the purposes of this review, principles were determined by the literature and not from discussions held with the expert panel, although their opinions and suggested modifications were sought in free text boxes during all iterative rounds.[18] The primary advantages of a modified Delphi technique include enhanced response rates, and a strong grounding in extant literature,[17] thus warranting its inclusion in the proposed research.

### 2.1 | Phase 1: Evidence review

To ensure transparency of findings and rigour, review findings follow the Centre for Reviews and Dissemination[19] and National Collaborating Centre for Methods and Tools guidance.[20] The peer review proposal is registered in the PROSPERO database No CRD42016035415.

#### 2.1.1 | Search strategy

To ensure sufficient coverage and be inclusive of the most up-to-date information, both peer-reviewed and grey literatures were reviewed.

The search terms listed below designed to maximize sensitivity and specificity were developed using the SPICE framework[21] and reviewed by both authors in line with the Peer Review of Electron Search Strategies (PRESS) guidance.[22]

##### 2.1.1.1 | Peer review

To avoid duplication of existing work, a systematic review of reviews published between 2010 and 2016 was conducted to assess how PPI is initiated and developed across the aforementioned settings. As advised by an information specialist (GT), one author searched Medline, EMBASE and PsycInfo for articles published in the English language. Due to limited resources, the authors could not ensure a sensitive interpretation of non-English articles and these were therefore excluded in both search strategies to maintain research integrity. Reference list and citation searches of eligible studies were also conducted. The search terms used to review peer-literature were as follows: "service users" OR "patient*" OR public OR lay AND involvement OR participation OR engagement OR co-production AND review.

##### 2.1.1.2 | Grey literature

For the purpose of this research, grey literature was defined as "that which is produced on all levels of governmental, academic, business and industry in print and electronic formats but which is not controlled by commercial publishers."[23] It was included on the basis that it would help validate the results of published literature searches, identify the most up-to-date information[24] and respond to criticisms of existing literature operating in silos.[13] A conventional search engine (Google) was used to identify grey literature using the following search terms: patient OR public OR "service user" AND involvement OR co-production OR participation OR engagement AND tool OR guide OR kit.

Review searches were conducted over a period of five days (01/03/2016 - 05/03/2016).

### 2.1.2 | Study selection

#### 2.1.2.1 | Peer review

Studies were selected through a two stage process. Two reviewers independently examined abstracts of identified studies for study inclusion using pre-defined inclusion criteria: systematic reviews published in the English language between 2010 and 2016; exploring PPI in medicine, dentistry or nursing across one or more of research, regulation, health-care services and educational settings. When an inclusion decision could not be made from the title and abstract alone, the full article was retrieved. Accepted studies were then read in full and independently assessed for study inclusion. Any discrepancies that could not be resolved by discussion were sent to a third reviewer for clarification until consensus was achieved.

#### 2.1.2.2 | Grey literature

Grey literature was assessed for inclusion by reading the titles and synopses of documents where available. Potentially relevant documents were obtained in full and either accepted or rejected on the grounds of irrelevance or failure to comply with pre-defined inclusion criteria: published in the English Language between 2010 and 2016; aimed at an identified population; and covered the aforementioned settings of interest. Due to the scale of grey literature, the inclusion of the words guide, toolkit or guidance in the title and UK only focus were used as a filter to maintain a manageable, yet extensive focus.

The peer-reviewed and grey literature selection process, including numerical values for included/excluded sources at each stage is shown in Figures 1 and 2.

### 2.1.3 | Data extraction

Two reviewers independently undertook data extraction using a piloted data extraction form. Information extracted included: year

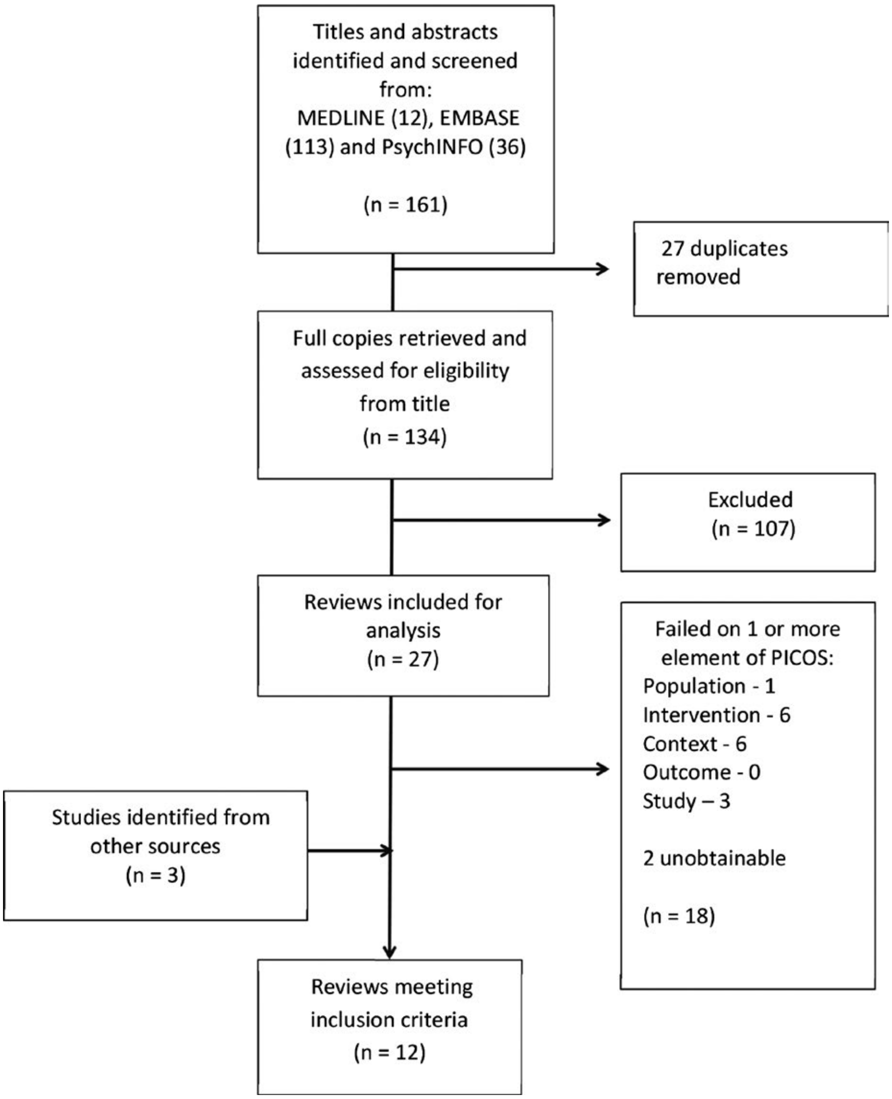

**FIGURE 1** Peer review selection process

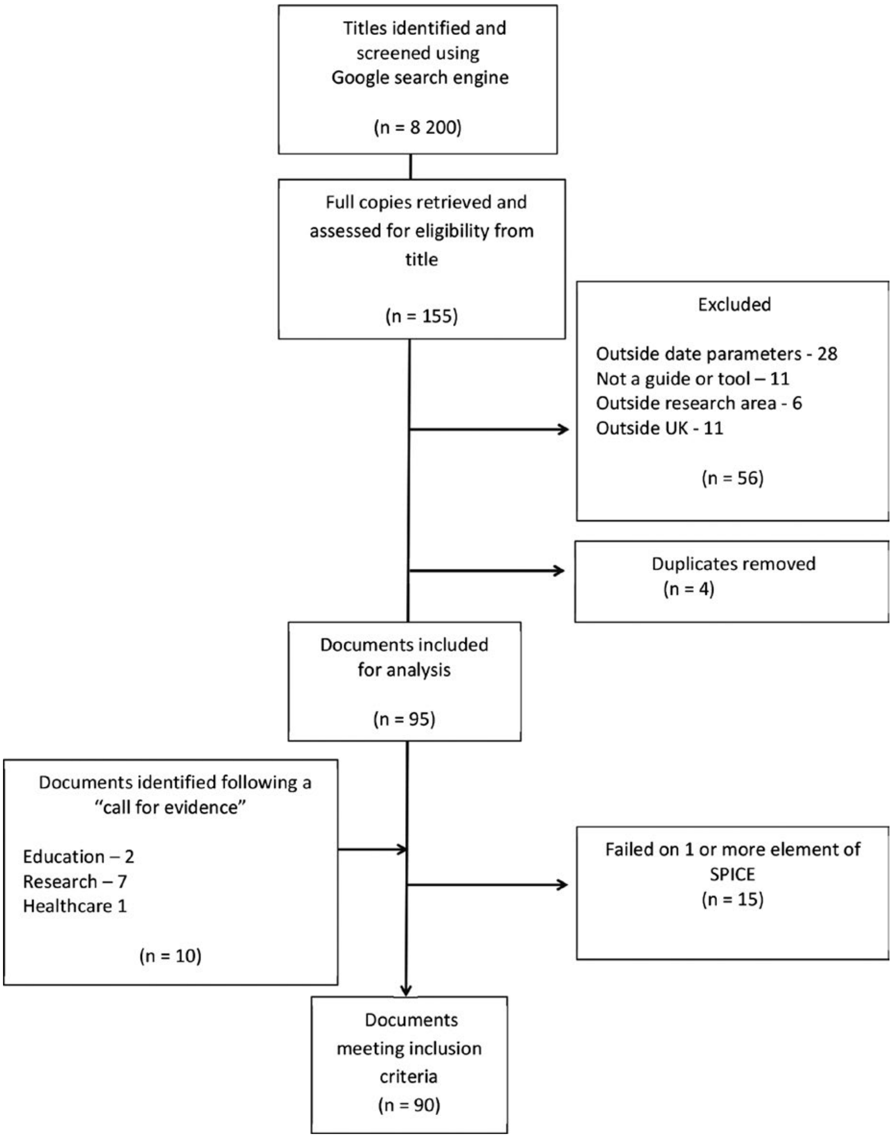

**FIGURE 2** Grey literature review process published; literature location, aim and design; study population and sample methodology; intervention type; and outcome findings.

#### 2.1.4 | Quality appraisal

Although not the focus of the review, included reviews were quality appraised independently using the Revised Assessment of Multiple Systematic Reviews (R-AMSTRA) measurement tool to ensure some reliability of the conclusions drawn. No formal quality assessment of the grey literature was undertaken as this was not the primary focus of the review.

### 2.2 | Phase 2: Thematic analysis

Review findings were thematically analysed using an inductive approach.[25] One researcher inductively analysed all content with a random 10% of documents analysed by a second researcher to ensure reliability.[25] No discrepancies or modifications were made to thematic analysis findings during this stage.

### 2.3 | Phase 3: Development and synthesis of PPI principles

Principles were inferred from the general themes identified in phase two. Inferred principles were developed based on content similarity and presence across the four areas of interest (research, regulation, education and health-care services). Principles that did not appear in all domains of interest were not developed further. Expert respondents were invited to suggest additional principles not identified and revise the structure and language of identified principles to ensure accessibility and understanding. Suggested modifications were made accordingly.

### 2.4 | Phase 4: Delphi technique

Defined as "a widely used and accepted method for achieving convergence of opinion concerning real-world knowledge",[26] the Delphi is regarded as a valid research technique applicable to a number of areas including evaluation, education, health and social care research.[15,18,26-28] Unlike surveys, the Delphi seeks to address "what

**TABLE 1** PPI principles rank ordered by expert respondents

| Principle identification | |
|---|---|
| 1 | Share information, experiences, knowledge and power |
| 2 | Listen, assess and respond to information shared. Regularly update people involved. Do not collect information and then ignore it as this is disrespectful and tokenistic. Act on information shared and offer clear explanations as to why suggested changes have not been acted on |
| 3 | Work in equal partnerships built on mutual trust, respect and transparency |
| 4 | Communicate and inform regularly, clearly and inclusively. Do not rely on one method of communication; this is unlikely to be suitable for all those involved—be creative |
| 5 | Support and prepare everyone involved before, during and after any *working together* initiative. This includes offering relevant training, information, practical, emotional and financial support. |
| 6 | Acknowledge, reward and value everyone involved. Celebrate good practice |
| 7 | Accommodate individual and collective needs to ensure inclusivity |
| 8 | Commit to *working together* on a personal, organisational and long-term basis |
| 9 | Be proactive in your approach. Go out into relevant communities and get involved. Do not expect people to come to you |
| 10 | Resource and invest. Effective *working together* takes time, money and resources. Be prepared to invest time and effort; it will nearly always take longer than you think |
| 11 | Empower all members involved. Ensure information, resources and skills are shared so everyone can contribute to decision-making processes |
| 12 | Tailor your approach, materials, training and evaluations provided to match your aim, purpose and local context |
| 13 | Evaluate throughout your *working together* initiative to identify best practice and areas that can be improved |

could/should be"[26] as opposed to "what is" through a series of iterative questionnaires on topics where consensus has previously not been achieved.[26,29] Its main benefits include the attainment of anonymous "expert" opinions without the time and geographical restraints experienced in alternative methods.[28]

However, since its inception, the Delphi has undergone a series of modifications with no universally agreed guidelines surrounding its appropriate design, "expert" definition or appropriate expert panel size.[15,18] Despite this, it is widely accepted that the appropriate use of a Delphi methodology requires a high degree of methodological precision and research rigour.[29]

For the purpose of this research, experts were defined as "those who are knowledgeable about, or skilful in, a particular area."[30] In this instance those with experience of PPI in health and social care services, research, education and/or regulation. Expert respondents were asked to:

Round 1: rank order principles in terms of importance based on their own experience to establish preliminary priorities amongst them.

Round 2: clarify or revise their judgement following a revised questionnaire built around the responses generated from Round 1.

Round 3: make any final comments or revisions following the sharing of ratings, and identification of minority, and majority opinions.

Results from rounds one and two were fed back to the experts in the form of line graphs and simple textual summaries of statistical data. The order in which identified principles were presented to expert respondents remained consistent. The possibility of creating an order effect is acknowledged.[31] All rounds were administered via email or posted if requested.

#### 2.4.1 | Sampling

Patient and public involvement experts identified through the social and professional networks of the research team were invited to take part through advertisements distributed through the Patients Association, Lived Experience Network and Developers of User and Carer Involvement in Education network.

In line with accepted guidance,[27] which suggests recruiting 10-18 participants, 22 experts were contacted to take part to allow for potential dropouts or non-inclusion.

#### 2.4.2 | Consensus definition

Consensus was defined as 75% or greater agreement. This is based on the results of a recent systematic review where percentage agreement was identified as the most common definition of consensus. 75% was the median threshold used to define consensus in a random selection of 100 studies.[32]

### Ethical approval

Ethical approval was obtained through the Faculty Research Ethics Committee for Health and Human Sciences at Plymouth University.

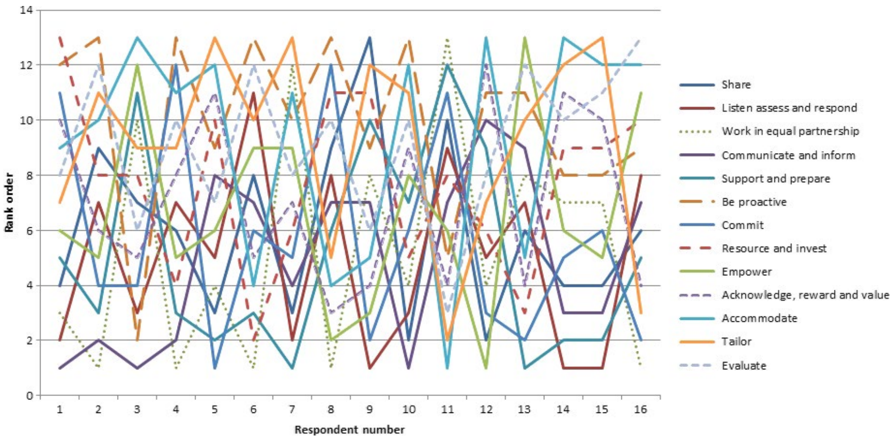

**FIGURE 3** Round 1: rank ordering of underlying principles

## 3 | RESULTS

Figures 1 and 2 demonstrate the selection process for the peer-reviewed and grey literature searches. A total of 12 peer-reviewed systematic reviews focusing on: medical education (n = 2); health-care research (n = 6), regulation (n = 1) and health-care services (n = 3) were included. One review was UK centric, the remaining 11 drew on international literature predominantly from the US, Canada, Australia and New Zealand. The overall quality of included reviews was mixed. 88 grey literature publications were included that identified themselves as: guidance (n = 15); guidelines (n = 22); guides (n = 33); tools (n = 5); toolkits (n = 10); handbooks (n = 1); or a catalogue (n = 1).

### 3.1 | Principle identification

Thematic analysis of review findings led to the identification of 13 principles listed in Table 1. These principles were then tested for consensus amongst an expert population.

### 3.2 | Respondents

A total of 21 experts responded, with three incomplete responses provided. Eighteen responses were therefore included for analysis. Respondents identified themselves as: service users or carers (n = 6); researchers (n = 4); lecturers or professors in nursing and/or midwifery (n = 3); public and patient manager/officer (n = 2); patient educator tutor (n = 1); director of teaching and learning (n = 1); and interprofessional education co-ordinator (n = 1).

### 3.3 | Round 1

No consensus was achieved at any rank order level when expert respondents were asked to rank order principles based on their own experience (Figure 3). The rank order value of two for working in equal partnership achieved the highest agreement rate of 55.5% (n = 10/18). All other rank orderings were highly variable.

Reflecting the ethos of co-production and feedback from expert respondents regarding the difficulty of this exercise, a collaborative decision was made to order principles into three clearly defined categories: those deemed necessary; desirable; or irrelevant. This decision was made on the basis of difficulties experienced in rank ordering principles and not the total number of principles included.

### 3.4 | Round 2

Of the original 18 respondents, 17 agreed to take part in the second Delphi round achieving a 94.1% response rate. Seven of the 13 principles achieved essential consensus (75%). The six principles that failed to achieve essential or desirable consensus were as follows: *empower* (10/17 essential, 58.8%; 7/17 desirable, 41.2%); *tailor* (11/17 essential, 64.7%; 6/17 desirable 35.3%); *commit* (11/17 essential 64.7%; 6/17 desirable, 35.3%); *evaluate* (12/17 essential, 70.6%; 5/17 desirable, 29.4%); *be proactive* (10/17 essential 58.8%; 7/17 desirable, 41.2%) and *resource and invest* (10/17 essential, 58.8%, 7/17 desirable 41.2%).

### 3.5 | Round 3

During round 3, a total of seven categorization alterations were made (listen, assess and respond n = 1 re-categorization; acknowledge n = 1; tailor n = 2; evaluate n = 1; resource and invest n = 2), leading to the re-categorization of two principles as essential (evaluate: 13/17 agree, 76.5%; and tailor 13/17 agree, 76.5%). Alterations made in this final stage are highlighted in bold in Table 2. Principles that failed to achieve essential consensus were as follows: *commit* (11/17 essential 64.7%; 6/17 desirable, 35.3%); *resource and invest* (11/17 essential 64.7%; 6/17 desirable, 35.3%); *empower* (10/17 essential, 58.8%; 7/17 desirable, 41.2%); *and be proactive* (10/17 essential 58.8%; 7/17 desirable, 41.2%).

Table 3 Outlines the final principle categorization and their subsequent gradation.

No alternative principles or suggested modifications were proposed by expert respondents at any stage.

## 4 | DISCUSSION

This research advances current understanding of PPI practice by identifying and evaluating its essential and desirable principles from both a

**TABLE 2** Rounds 2 and 3 Delphi categorization percentages

| | Round 2 | | Round 3 | |
|---|---|---|---|---|
| **Principle** | **Essential** | **Desirable** | **Essential** | **Desirable** |
| *Work in equal partnerships* | 94.1% | 5.9% | 94.1% | 5.9% |
| *Share* information, good practice, negative experiences, knowledge and power | 94.1% | 5.9% | 94.1% | 5.9% |
| *Communicate and inform* | 88.2% | 11.8% | 88.2% | 11.8% |
| *Listen, assess and respond* | 82.4% | 17.6% | **88.2%** | **11.8%** |
| *Support and prepare* | 82.4% | 17.6% | 82.4% | 17.6% |
| *Acknowledge, reward and value* everyone involved | 76.5% | 23.5% | **82.4%** | **17.6%** |
| *Accommodate* individual and collective needs | 76.5% | 23.5% | 76.5% | 23.5% |
| *Evaluate* throughout | 70.6% | 29.4% | **76.5%** | **23.5%** |
| *Tailor* your working together approach | 64.7% | 35.3% | **76.5%** | **23.5%** |
| *Commit* to working together | 64.7% | 35.3% | 64.7% | 35.3% |
| *Resource and invest* | 58.8% | 41.2% | **64.7%** | **35.3%** |
| *Empower* all members involved. | 58.8% | 41.2% | 58.8% | 41.2% |
| *Be proactive* in your approach. | 58.8% | 41.2% | 58.8% | 41.2% |

>75% agreement = essential consensus.
Numbers in bold indicate a re-categorization.

patient and professional perspective. It addresses Brett et als' call for additional research into this topic area and provides previously unavailable consensus about how best to optimize PPI grounded in the findings of international literature.[11,14] The research contributes new knowledge in several ways. Firstly, literature surrounding PPI has typically been fragmented, and this research is the first of its kind to draw on a systematic review that unites these fragmented literatures into an overarching narrative about its purpose and underpinning principles. Secondly, previous research has typically explored what might be counted as effective PPI from the perspective of professionals or regulators only. The innovative methodology employed in this research has enabled patients and carers to be direct contributors to the outcome and direction of this research, its subsequent publication and potential impact.

The lack of consensus reported in the first Delphi round is perhaps to be expected, given the complexity of PPI reported in existing literature.[11] In particular, available evidence highlights the influence different contexts have on the appropriate forms and functions of PPI.[14] For example, those working in nursing may rank principles differently from those working in dentistry because they have different agendas to address. In acknowledgement of this, a collaborative decision was made by both the research team and the expert panel to categorize proposed principles into three distinct categories: essential, desirable, or irrelevant.

Inevitably, principles that achieved majority consensus were often those that provided solutions to the most frequently cited barriers of effective PPI.[14,15,33] For example, one frequently identified barrier is the unequal power dynamics caused by the paternalistic relations that often exist between clinicians and patients.[33] The principle of working in equal partnerships identified in the review is a direct solution to this. Other examples of this parallel relationship include listening, assessing and responding to the information shared to prevent a sense of abandonment or lack of change, a further problematic experience of ineffective PPI.[14,15,33]

Principles that failed to achieve "essential" consensus and were therefore categorized as "desirable" are described by expert respondents as principles that can "transcend" mere compliance to PPI agendas. One expert respondent wrote "*those* [desirable principles] *aspects may not always be achieved hence they can only be desirable. By comparison, those I considered essential are attainable.*" This distinction between the *desire* for all principles to be achieved, and the *reality* of achieving them is worth noting, as PPI is contingent on available resources, suitable conditions and buy-in from all those involved.[14,15,33]

One important distinction to make is that the principles suggested provide quality guidelines for best practice, not prescriptive rules. The proposal of a "one size fits all" approach to PPI would be inappropriate, as no single PPI initiative will work for all situations, individuals or agendas. However, our research suggests that whilst PPI must be adaptable to local circumstances and objectives, the essential principles required to underpin its effectiveness may well be universal in their application.

## 4.1 | Strengths and limitations

Strengths of this research include its application of a recognized systematic review process[19] and inclusion of a large sample of both peer-reviewed and grey literatures. Grey literature is often excluded from systematic reviews due to its associated searching difficulties.[24,34]

**TABLE 3** Final categorization of essential and desirable PPI principles

| |
|---|
| **Essential** |
| *Work in equal partnerships* built on mutual trust, respect and transparency |
| *Share* information, good practice, negative experiences, knowledge and power |
| *Communicate and inform* regularly, clearly and inclusively. Do not rely on 1 method of communication; this is unlikely to be suitable for all those involved—be creative |
| *Listen, assess and respond* to the information shared. Regularly update people involved. Do not collect information and then ignore it. This is disrespectful and tokenistic |
| *Support and prepare* everyone involved before, during and after any working together initiative. This includes offering relevant training, information, practical, emotional and financial support |
| *Acknowledge, reward and value* everyone involved. Celebrate good practice |
| *Accommodate* individual and collective needs to ensure inclusivity |
| *Evaluate* throughout your working together initiative to identify best practice and areas that can be improved |
| *Tailor* your working together approach, materials, training and evaluations provided to match your purpose, aim, local context and individual choices of people involved |
| **Desirable** |
| *Commit* to working together on a personal, organisational and long-term basis. Do not involve sporadically, be consistent |
| *Resource and invest*: Effective working together takes time, money and resources. Be prepared to invest time and effort in working together; it will nearly always take longer than you think |
| *Empower* all members involved |
| *Be proactive* in your approach. Do not expect people to come to you. Go out into relevant communities, get involved |

However, such literature often includes the most up-to-date information from those "working" on the ground, and thus, in turn, presents alternative perceptions to those available in peer-reviewed literature.[35] For this research, grey literature provided more practical approaches to PPI and typically moved beyond issues of semantic intricacies largely discussed in the peer-reviewed literature. The integration of typically disparate literatures into one readily accessible corpus of information is also a recognized strength of this study. As a result, research findings will be applicable to the multiple areas investigated including health and social care services, research, education and regulation across medicine, dentistry and nursing demonstrating widespread transferability. Furthermore, whilst potentially not exhaustive, all 13 principles were considered relevant by expert respondents, with no alternative principles suggested, demonstrating high levels of relevance, suitability and coverage. This may be indicative of the extensive review process and adoption of inductive thematic analysis avoiding the use of pre-defined frameworks. Finally, the innovative application of a systematic review and modified Delphi methodology is the first of its kind in this area.

However, as with all research, some limitations should also be acknowledged. The majority of expert respondents were UK based. However, the international focus of the literature review from which the principles were constructed ensures a certain level of transferability. The authors also acknowledge that the sample size of 18 is not exhaustive. However, the Delphi methodology rests on the assumption that group opinion carries greater validity than that of one individual and is, therefore, concerned with group dynamics as opposed to extensive statistical power.[15,35] Current literature recommends 10-18 expert respondents.[27] A recent review of 100 published Delphi studies found 40% of all studies reviewed had 11-25 respondents.[32] The sample size in this research, therefore, follows existing recommendations and is in line with current practice.

Expert respondents were also purposively sampled volunteers, and the potential for bias in this approach is acknowledged.[18] However, given the requirement for experts to have subject knowledge, expert respondents could not be selected at random.[18] Hasson et al suggest the involvement of individuals with subject knowledge may enhance the content validity of Delphi findings, and the use of successive rounds may also increase concurrent validity.[29] The validity of these results should not therefore be undermined. Finally, response exhaustion can be an issue when using the Delphi method.[18] Although one respondent did not participate in rounds 2 and 3, the response rate of 94.1% achieved in this research is highly favourable.

### 4.2 | Implications for practice

This research holds important practical and theoretical implications for policy makers, patients, carers and professionals wishing to work together across the continuum of health and social care. Principles that are considered essential must be present for PPI to be effective. Principles identified as desirable must also be aspired to if PPI practice is to "transcend" mere lip service to mandatory PPI agendas. From a theoretical perspective, this research is the first of its kind to bridge the disparate corpus of PPI knowledge across a multitude of disciplines and topic areas leading to the co-production of a principle taxonomy pertinent to all those looking to work together.

## 5 | CONCLUSION

This research uniquely identifies and evaluates the principles underpinning effective PPI from both an academic and patient expert perspective. In doing so, it advances existing understanding by enhancing academic and practical understanding of how best to optimize PPI. It provides previously unavailable consensus about PPI best practice applicable to the multiple areas of interest studied.

### ACKNOWLEDGEMENTS

The authors would like to thank the following individuals for their participation: Jill Anderson; Michael Ashman; Sue Baron; Arthur Carr; Chris Essen; Shelley Fielden; Ray Jones; Mirza Lalani; Sol Mead; Alison Morris; Hugh Norman; Christine Raw; Dr Christine Rhodes; Laura Sherlock; Rosamund Snow; Sebastian Stevens; Tony Summer; Katie

Swinburn; Jools Symons; Sally Thomasson; Lyndsey Withers; Kim Young; the Patients Association; members of the Lived Experience Network (LEN) and The Developers of User and Carer Involvement in Education networks (DUCIE). Their time and insights have been invaluable. We would like to thank co-applicant Dr Suzanne Nunn FRSA who designed and undertook the review of reviews (phase 1 of the research). Finally, this article is dedicated to the memory of Rosamund Snow, an inspirational woman in every way.

## CONFLICTS OF INTEREST

No conflicts of interest have been declared.
